# Meetings of Societies

**Published:** 1930

**Authors:** 


### Meetings of Societies.

### Bristol Medico-Chirurgical Society.

The second meeting of the session was held at the University on Wednesday, 13th November, 1929, the President in the chair.

Mr. E. Watson-Williams read a paper on "**Cancer of the Tonsil.**" *Sarcoma* accounted for about 20 per cent. of cases. Two out of three patients were males, and the disease might occur at any period of life, even in infancy, though most frequent in middle age. Spindle-cell, round cell, mixed, myxo- and melanotic sarcoma and endothelioma are described. Clinically the two groups of fibrosarcoma and lymphosarcoma are recognized. Attempts at classification by tissues of origin have been made: as, fibrosarcoma arising from vascular and connective tissue, reticulosarcoma arising from gland reticulum and true lymphosarcoma or lymphocytoma malignum from the lymph cells of the tonsil itself. Such a classification is premature; the different types merge one into another even in the course of a single case, and a purely clinical classification is probably as useful as any.

*Fibrosarcoma* is a condition of local malignancy, involving surrounding structures, but not producing glandular metastases.

The first complaint is of a lump in the throat or of consequent difficulty in speaking or swallowing. One tonsil is found to be enlarged or to be replaced by a large red or purple mass in the faucial pillar. Ulceration is at first absent. Extension occurs into the deep tissues of the neck, and an external swelling appears behind and fixed to the ascending ramus of the jaw. In the later stage ulceration and sloughing occur, and distant metastases are found in liver, etc.

*Lymphosarcoma* resembles the above in general features, but there is early involvement of the cervical glands : this is said to be by lymphatic permeation and not by embolic metastasis, and affects both sides of the neck even from one tonsil. Extension later occurs to other glands, especially in the mediastinum. These glands are hard and fixed, and tend to coalesce and to ulcerate.

*Lymphocytoma malignum* represents the most malignant variety. The condition is described by Kundrat as affecting healthy middle-aged males, and starting either in a group of glands or in the lymph tissue of the alimentary canal. The tonsil provides one of the most important groups of this condition. Both tonsils may be involved simultaneously or in swift succession ; glandular affection is early and extensive. The glands remain firm, discrete and mobile, and many groups of glands all over the body may show changes ; blood changes are common. Kundrat considered tuberculosis played no part in the condition, but others have held the contrary view.

Diagnosis rests on the clinical appearances, and is confirmed by histological examination. In many of the reported cases an incision has been made in search of pus ; the absence of œdema, pain and fever should prevent this mistake. Treatment is unsatisfactory. Where there is no involvement of glands operative removal may be successful (? 10 per cent.), and an operation through the mouth offers as good a prospect as lateral pharyngotomy with removal of glands and division of mandible. These growths are very susceptible to radium and X-rays, but in only too many cases the condition recurs and proves intractable.

[The speaker then described three cases of sarcoma of the tonsil under his care.]

*Carcinoma* of the tonsil is a disease of later life, rare before 45 ; seven out of eight patients are males. Rare examples of basal-cell and of adeno-carcinoma have been seen, but the common type is squamous epithelioma. Clinically two types may be recognized, the acute and the slow. In the former there is complaint of pain on swallowing, pain in the ear, or sore throat worse at night, with salivation, fetor of breath and rapid wasting. Within five or six weeks of the onset ulceration, obviously malignant, has involved a large part of the fauces, and may already have spread to the opposite side, out on to the cheek, into the base of the tongue, or the posterior pharyngeal wall. In the very acute cases the patient might be obviously dying before glandular involvement was apparent. The slow type was in its later phases prone to transition to the rapid, and one might consider here those cases which came with a history of many months of trouble, and with similar very extensive ulceration, but as a rule with very greatly enlarged cervical glands in addition. Of the speaker's cases four belonged to the former and five to the latter class. Treatment is almost impossible here, although if not too advanced radium produces a temporary alleviation. In three cases selenium injection was followed by a few weeks' remission of symptoms. Orthoform is generally advised, but as a rule opium is the only effective anodyne.

In the slower type the earliest symptoms are: (1) Pain in the ear, often worse at night; *or* (2) pain in the throat, worse on swallowing, or the sensation of a lump, with a constant desire to swallow; *or* (3) enlarged glands in the neck.

In the early stage a hard nodular growth is seen or felt on the tonsil or just at the edge. It soon ulcerates, and then presents the hard everted edge so characteristic of malignancy. As has just been said, the cervical glands are early involved.

Cases cited exemplify each of these modes of onset, and the extreme importance of early recognition. The most frequent mistake is to waste valuable time on dental treatment; gross dental sepsis is very commonly associated with malignant disease of the pharynx and œsophagus, but does not produce painless, hard, fixed cervical adenitis. Laboratory tests should not be relied on to exclude carcinoma; a syphilitic scar can become the site of malignant disease, as in a case cited; many malignant ulcers would yield Vincent's spirillum. But a gummatous ulcer was seldom very painful, and would be healing rapidly at the end of a week on intensive iodide medication, just as Vincent's angina yielded to arsenic. In every case where there was any doubt at all a biopsy should be at once performed, and would generally settle the diagnosis, except that where all treatment is declined it is better to avoid even a biopsy.

[The speaker then related case histories illustrating the mistakes that could be avoided.]

Operation should be attempted whenever possible. Suitable cases are those in which the primary growth was confined to the tonsil, or at least did not invade the tongue, and in which the glands were not so fixed that the internal carotid was probably involved. Lateral pharyngotomy is a severe operation and probably unnecessary. The operation of choice is carried out in two stages: (1) Block dissection of the neck glands, etc. (with ligature of the external carotid); (2) diathermy dissection of the affected tonsil and surroundings. Radiation therapy is possibly advisable after the operation.

In cases unsuitable for operation the implantation of radium needles around the growth has met with a fair measure of success; in these, however, the problem of the neck glands is always difficult. The gland part of the operation is the serious stage, and radiation of glands has not met with a large measure of success. In conjunction with other methods, or even alone, injections of colloidal selenium are of the greatest value.

[The speaker then described the five cases shown before the meeting, three treated by operation, one by selenium and radium, and one by selenium alone—all apparently "cured," though one is very recent. He referred to palliative operations and their results at his hands. He mentioned a number of cases in which recurrence had taken place, to emphasize the frequency with which the tongue was invaded. Finally, he uttered a plea for early recognition of these cases as the necessary precursor of effective treatment. Epidiascopic illustrations of early appearances were given.]

### DISCUSSION.

The PRESIDENT asked whether Mr. Watson-Williams had been troubled by secondary hemorrhage after diathermy, and whether he found that much shock resulted from a block dissection, and referred to cases where very large glands had been present at a time when the primary growth was of insignificant size.

MR. A. J. WRIGHT was glad the speaker had emphasized the importance of not assigning dental sepsis as a cause of

G

enlarged cervical glands. He asked whether he had seen a case in which growth began in a tonsillar crypt out of sight.

Mr. W. A. Jackman gave details of one of the cases shown, the man treated with selenium only, and said he was very glad so good a result had been obtained.

Dr. P. Watson-Williams emphasized the need of early diagnosis in these cases, and said that he felt treatment with selenium had not been everywhere given an adequate trial.

Dr. Sutherland inquired whether a swab report would not serve more rapidly than medication to establish a diagnosis of Vincent's angina.

[Answer.—Quite, but a positive report does not *exclude* carcinoma.]

---

The third meeting of the session was held on 11th December, 1929, when Mr. A. Rendle Short made a report on "**Some Experiences with Radium in the Treatment of Cancer.**" He pointed out that the great improvement which has taken place in the results is due to the alteration of technique, in the direction of using small doses for a week, instead of large doses for a day. This report must be regarded as very tentative, because our experience is so recent, and the quantity of radium at present available at public institutions and in private hands in the town is far too small for the needs of the district.

He presented a report on twenty-one cases, which included all treated, except a few in which radium was used merely as an adjunct to surgery. Of the twenty cases, four were considered operable, seventeen inoperable. The results were as follows: Two died; four little, or no better; three resulted in disappearance of growth locally, but now suffer from metastases; six gave good results, and are alive and free from recurrence; six others were improved, but results must be classed as doubtful. (Since the report one of these has definitely recurred, and another seems to be clinically cancer free.)

The regional distribution of cases was as follows: Prostate two, both improved; rectum five, one did well; breast four, all did well; mouth and tongue six, five improved, but three recurred.

The fourth meeting of the session was held on 8th January, 1930. DR. A. T. TODD read a paper on "**Dyspepsia.**" The current theories of pain production were reviewed. It was pointed out that dyspeptics could be divided into two classes, *i.e.* those complaining of pain and those who had no pain. It was shown that the dyspeptics who complained of pain had hyperalgesia of certain muscles of respiration and that pressure on these muscles reproduced the pain felt after food. It was shown that there was also respiratory disability and that shortness of breath was not infrequent.

The dyspeptics who did not complain of pain did not show this hyperalgesia and were not short of breath.

The importance of the respiratory pump action on the circulation was discussed ; failure of this action intensified the shortness of breath produced by the respiratory disability.

It was shown that all graduations from this simple pain, through the effort syndrome to fully-fledged angina pectoris were to be found in painful dyspepsia. That adequate treatment of this group of anginal cases was followed by disappearance of the symptom.

It was concluded that gastric pain was the result of a reflex spasm of certain respiratory muscles. That shortness of breath was frequently produced and then the condition was wrongly diagnosed as cardiac. That the pain might be that of angina pectoris and a possible result of continued muscular irritation causing arterial degeneration was demonstrated.

---

The fifth meeting of the session was held at the University on Wednesday, 12th February, 1930, the President in the chair. PROFESSOR D. P. D. WILKIE gave an address on "**Acute Intestinal Obstruction,**" which will appear in our next issue.

